# Segurança e Eficácia da Terapia com Células-tronco Mesenquimais Derivadas do Tecido Adiposo para Cardiopatia Isquêmica: Revisão Sistemática

**DOI:** 10.36660/abc.20230830

**Published:** 2024-09-06

**Authors:** Fernando Rabioglio Giugni, Melina de Oliveira Valdo Giugni, Henrique Trombini Pinesi, Fabio Cetinic Habrum, Lígia Nasi Laranjeira, Erica Regina Ribeiro Sady, Erica Aranha Suzumura, Luis Henrique Wolff Gowdak, José Eduardo Krieger

**Affiliations:** 1 The University of Texas Southwestern Medical Center Dallas Texas EUA The University of Texas Southwestern Medical Center, Dallas, Texas – EUA; 2 Hospital das Clinicas Faculdade de Medicina Universidade de São Paulo São Paulo SP Brasil Instituto do Coração InCor, Hospital das Clinicas HCFMUSP, Faculdade de Medicina, Universidade de São Paulo, São Paulo, SP – Brasil; 3 Baylor University Medical Center at Dallas Dallas Texas EUA Baylor University Medical Center at Dallas, Dallas, Texas – EUA; 4 Hospital do Coração São Paulo SP Brasil Hospital do Coração (HCor), São Paulo, SP – Brasil; 5 Departmento de Medicina Preventiva Faculdade de Medicina Universidade de São Paulo São Paulo SP Brasil Departmento de Medicina Preventiva, Faculdade de Medicina FMUSP, Universidade de São Paulo, São Paulo, SP – Brasil

**Keywords:** Células-Tronco Mesenquimais, Medicina Regenerativa, Isquemia Miocárdica, Terapia Baseada em Transplante de Células e Tecidos

## Abstract

**Fundamento:**

A terapia celular utilizando células-tronco mesenquimais derivadas do tecido adiposo (ADSC, sigla em inglês) apresenta grande potencial como tratamento para doenças cardiovasculares.

**Objetivo:**

Realizamos uma revisão sistemática para descrever a segurança e a eficácia das ADSC na cardiopatia isquêmica.

**Métodos:**

Pesquisamos na PubMed/MEDLINE, EMBASE, Web of Science, CENTRAL e LILACS (desde o início até março de 2024) por estudos clínicos envolvendo ADSC em pacientes com cardiopatia isquêmica. Excluímos estudos envolvendo pacientes com outros tipos de doenças cardíacas, estudos utilizando células-tronco mesenquimais derivadas de outros tecidos, bem como estudos em andamento. Dois revisores independentes realizaram a triagem das citações recuperadas, extraíram dados relevantes e avaliaram o risco de viés nos ensaios incluídos, utilizando os critérios da Colaboração Cochrane modificados pela Universidade McMaster e o Índice Metodológico para Estudos Não-Randomizados (MINORS). Utilizamos uma síntese narrativa para apresentar os resultados.

**Resultados:**

Dez estudos (compreendendo 29 publicações) preencheram nossos critérios de inclusão, incluindo 8 ensaios controlados randomizados e 2 ensaios não controlados. Não foram relatados eventos adversos graves associados à terapia com ADSC. Embora a maioria dos desfechos de eficácia não tenha alcançado significância estatística, houve relatos de melhora da área isquêmica, capacidade funcional, sintomas e contratilidade em pacientes tratados com ADSC.

**Conclusões:**

Os resultados da nossa revisão sugerem que a terapia com ADSC é geralmente segura para pacientes com cardiopatia isquêmica. Contudo, são necessárias mais investigações para confirmar a sua eficácia, particularmente em ensaios clínicos de maior escala e em condições específicas onde as melhorias na microcirculação podem ter um impacto notável nos desfechos clínicos.

## Graphical abstract

**Figura Central:**
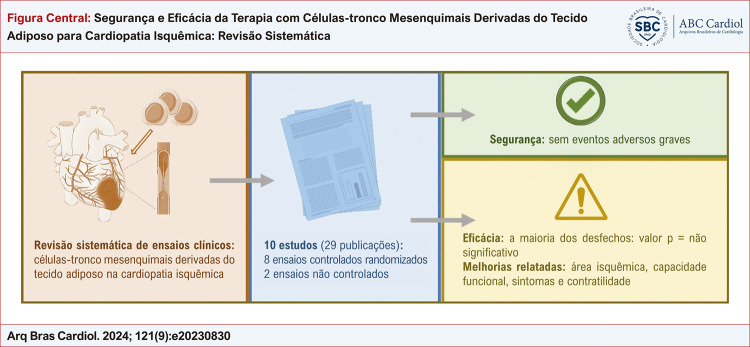
: Segurança e Eficácia da Terapia com Células-tronco Mesenquimais Derivadas do Tecido Adiposo para Cardiopatia Isquêmica: Revisão Sistemática

## Introdução

Nas últimas duas décadas, a terapia com células-tronco surgiu como uma abordagem promissora para o tratamento de diversas condições que têm respostas limitadas ou que não respondem às terapias convencionais. Inicialmente, o foco estava na capacidade regenerativa das células-tronco, na sua capacidade de autorrenovação e de diferenciação em diversos tipos de células.^1-3^ No entanto, evidências recentes sugerem que os efeitos terapêuticos da terapia com células-tronco são mediados principalmente por fatores parácrinos, que modulam a resposta natural do corpo a lesões, tanto agudas quanto crônicas.^4,5^

Extensos estudos *in vitro* caracterizaram vários tipos de células progenitoras, e modelos animais mostraram resultados promissores na avaliação da eficácia da terapia com células-tronco para diferentes condições. Esse progresso abriu caminho para os primeiros ensaios clínicos envolvendo a utilização de células-tronco adultas autólogas ou alogênicas. Diversos tipos de células-tronco adultas têm sido investigados, incluindo mioblastos esqueléticos, células derivadas da medula óssea, células-tronco cardíacas, células progenitoras endoteliais derivadas do sangue e células-tronco derivadas do tecido adiposo (ADSC, sigla em inglês).^2,6-9^

O tecido adiposo, que se origina do mesênquima embrionário, fornece uma fonte facilmente acessível de células estromais.^10^ As ADSC podem ser isoladas de resíduos de lipoaspiração humana após tratamento com colagenase e centrifugação. Semelhante a outras células-tronco mesenquimais, as ADSC podem ser induzidas a se diferenciar em vários subtipos celulares *in vitro* pela modificação do meio de cultura celular com fatores específicos.^11^ Devido à sua natureza versátil, as ADSC têm sido extensivamente estudadas no campo da medicina regenerativa, com aplicações variando de feridas crônicas na pele e defeitos de tecidos moles a doenças inflamatórias intestinais, diabetes mellitus tipo 1, lesões da medula espinal e acidente vascular cerebral.^12-16^

Sob condições experimentais específicas, as ADSC também podem se diferenciar em células do sistema cardiovascular.^17,18^ Além disso, as ADSC liberam fatores parácrinos que modulam as propriedades do microambiente tecidual.^5,19^ Esses fatores promovem a neovascularização, reduzem a apoptose e a inflamação e inibem a fibrose, melhorando assim o reparo cardíaco e a recuperação funcional. Estudos pré-clínicos forneceram evidências substanciais que apoiam o potencial das ADSC para reparo cardíaco em humanos.^7,20,21^ Adicionalmente, as ADSC podem ser obtidas em grandes quantidades e expandidas para uso terapêutico futuro, o que é vantajoso para terapias baseadas em células.^11^

Pacientes com doença arterial coronariana avançada que apresentam angina refratária ou insuficiência cardíaca isquêmica representam um desafio clínico significativo. Os medicamentos antianginosos podem não aliviar adequadamente os sintomas, e os procedimentos de revascularização miocárdica podem não ser viáveis devido ao fluxo arterial distal inadequado ou à doença obstrutiva aterosclerótica difusa, entre outras razões.^22^ Para alguns indivíduos com insuficiência cardíaca isquêmica progressiva, o transplante cardíaco torna-se a única opção viável para melhorar a sobrevida e a qualidade de vida apesar do tratamento farmacológico otimizado.^23^ Nessas situações, o tratamento com ADSC poderia servir como uma estratégia terapêutica alternativa, visando aumentar a neo-angio/vasculogênese, melhorar a disfunção endotelial e reduzir a inflamação e a fibrose, denominadas coletivamente como reparo cardíaco. Contudo, a segurança e eficácia da terapia com ADSC para essas condições ainda estão sendo investigadas.

Para melhor conhecer o estado atual da terapia com ADSC para cardiopatia isquêmica, realizamos uma revisão sistemática de estudos clínicos (Figura Central). Nosso objetivo foi identificar as lacunas de conhecimento existentes e as áreas que requerem investigação adicional para avançar nesta abordagem terapêutica.

## Métodos

A presente revisão sistemática seguiu as diretrizes recomendadas pela Colaboração Cochrane,^24,25^ e os resultados foram relatados seguindo os Principais Itens para Relatar Revisões Sistemáticas e Metanálises (PRISMA).^26^

### Fontes de dados de pesquisas

Realizamos pesquisas abrangentes nas seguintes bases de dados eletrônicas (desde o início até março de 2024): PubMed via MEDLINE, EMBASE, Web of Science, Cochrane Library/CENTRAL e LILACS. Não foram aplicadas restrições de idioma e, sempre que possível, foi utilizado vocabulário controlado (termos MeSH para MEDLINE e CENTRAL, EMTREE para EMBASE e DeCS para LILACS). Para aprimorar a estratégia de busca, utilizamos palavras-chave e seus sinônimos. A estratégia de busca completa é relatada na Tabela Suplementar 1. Adicionalmente, pesquisamos manualmente as listas de referências dos estudos incluídos para identificar outros artigos relevantes.

### Critérios de elegibilidade

Incluímos estudos que preencheram os seguintes critérios: (1) textos publicados na íntegra com os seguintes desenhos de pesquisa: ensaios randomizados ou quase randomizados, estudos observacionais comparativos ou série de casos não comparativos envolvendo pelo menos 10 pacientes; (2) estudos envolvendo pacientes com cardiopatia isquêmica aguda ou crônica; (3) avaliação dos efeitos do transplante de ADSC; (4) relato de pelo menos um dos desfechos de interesse. Excluímos estudos envolvendo pacientes com outros tipos de cardiopatia ou isquemia em outros órgãos que não o coração (por exemplo, periférico, cerebral, renal); estudos utilizando células-tronco mesenquimais derivadas de outros tecidos, como medula óssea, cordão umbilical, tecido sinovial ou sangue periférico; bem como estudos em andamento.

### Seleção de estudos

Dois revisores realizaram independentemente a triagem dos títulos e resumos de todas as citações recuperadas. Quando pelo menos um revisor considerava uma citação potencialmente adequada, a publicação em texto completo era obtida e avaliada em detalhes para confirmar a elegibilidade. Nos casos em que os estudos selecionados foram publicados em múltiplos periódicos (múltiplas publicações) ou incluíram subestudos, os dados foram listados na referência primária para fornecer informações adicionais. Estudos publicados apenas como resumos de conferências foram considerados inelegíveis devido a informações limitadas. As divergências entre os revisores foram resolvidas por meio de discussão, consenso ou consulta a um terceiro revisor.

### Extração de dados e avaliação de risco de viés

Dois revisores independentemente extraíram dados de estudos elegíveis usando um formulário padronizado e avaliaram o risco de viés com base em critérios específicos de domínio. Para ensaios randomizados ou quase randomizados, foram empregados os critérios da Colaboração Cochrane^25^ modificados pela McMaster University.^27^ Os estudos observacionais foram avaliados usando a ferramenta risco de viés de estudos de intervenção não randomizados (ROBINS-I, do inglês *Risk of Bias Summary for Non-randomized Studies*),^28^ enquanto os estudos não comparativos foram avaliados usando o Methodological Index for Non-Randomized Studies (MINORS, do inglês *Methodological Index for Non-Randomized Studies*).^29^ As divergências entre os revisores foram resolvidas por meio de discussão, consenso ou consulta a um terceiro revisor.

### Desfechos

Os desfechos de eficácia de interesse incluíram biópsia miocárdica, neovascularização colateral (angiografia coronária), classificação de angina da *Canadian Cardiovascular Society* (CCS), desempenho no teste de exercício (equivalentes metabólicos [METs], tempo de tolerância, carga [watts]), perfusão miocárdica (cintilografia miocárdica, ressonância magnética [RM], ecocardiograma de estresse, tomografia por emissão de pósitrons [PET] cardíaca) e viabilidade miocárdica (cintilografia miocárdica, ressonância magnética, ecocardiograma de estresse, tomografia por emissão de pósitrons [PET] cardíaca). Em pacientes com isquemia miocárdica e insuficiência cardíaca pré-intervenção, os desfechos adicionais foram a classificação funcional da New York Heart Association (NYHA) e a fração de ejeção do ventrículo esquerdo (FEVE). Foram coletadas todas as medidas de efeito de cada desfecho.

Os desfechos de segurança foram registrados como principais eventos adversos relatados nos estudos primários.

### Síntese de dados

Realizamos uma síntese narrativa dos resultados seguindo as diretrizes do Conselho Europeu de Pesquisa Social sobre a Conduta de Síntese Narrativa em Revisões Sistemáticas^30^ para responder às nossas questões de revisão. Os resultados relativos às características dos estudos incluídos, pacientes e intervenções utilizadas, bem como desfechos de eficácia e segurança, foram apresentados em tabelas de evidências.

## Resultados

**Figura 1:**
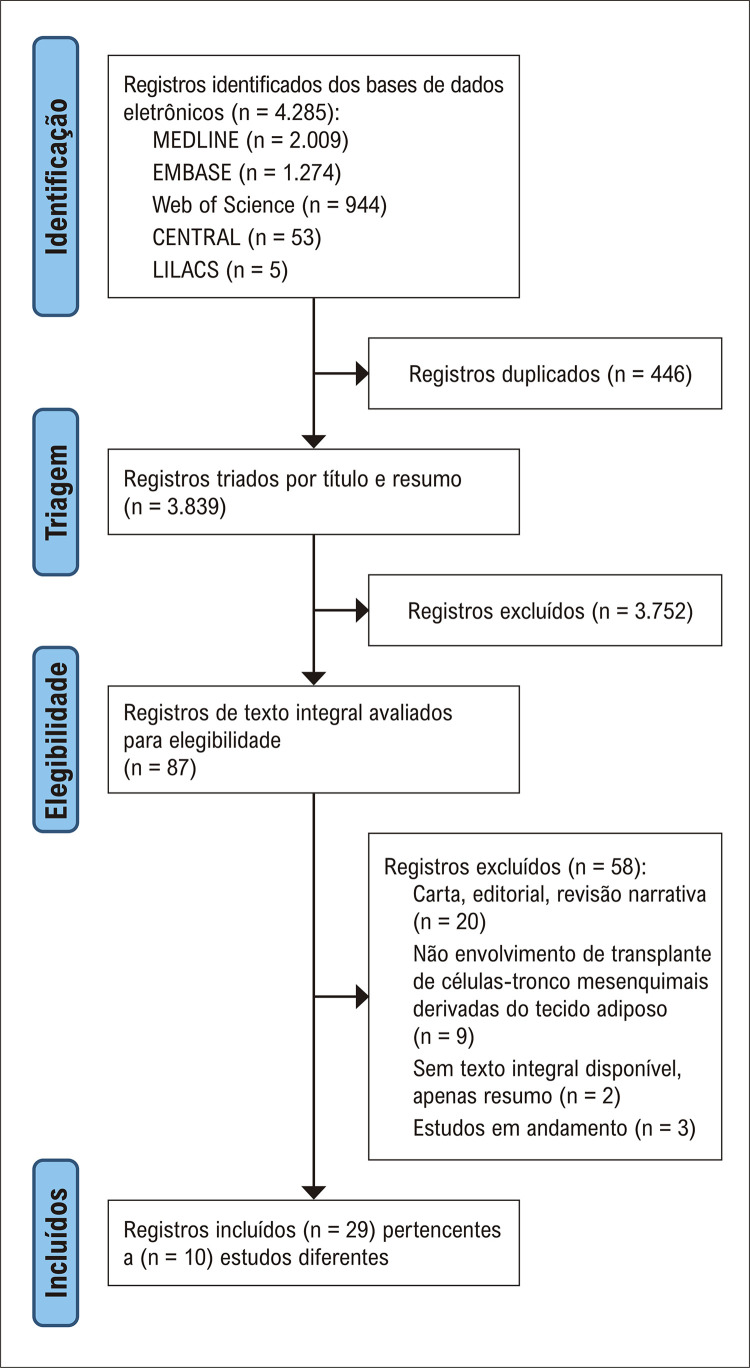
– Fluxograma de busca e seleção dos estudos.

A estratégia de busca rendeu 4.285 citações, das quais 446 foram excluídas por duplicação. Após a triagem dos títulos e resumos, foram revisadas 3.839 citações. Dentre elas, 87 citações relevantes foram selecionadas para posterior análise por meio da leitura das publicações completas. Posteriormente, 58 publicações foram excluídas por não preencherem todos os critérios de elegibilidade da presente revisão sistemática. Os motivos para a exclusão dos artigos após a revisão das publicações completas estão ilustrados na Figura 1. Por fim, foram incluídos 10 estudos (compreendendo 29 publicações) que avaliaram a segurança e a eficácia do transplante de ADSC para neo-angio/vasculogênese miocárdica em pacientes com cardiopatia isquêmica aguda ou crônica.^31-46^ O fluxograma de busca e seleção dos estudos é apresentado na Figura 1.

### Características dos estudos incluídos

A análise compreendeu 8 estudos randomizados^31-33,35,38,44,46^ e 2 estudos não controlados.^39,43^ publicados entre 2012 e 2023. A maioria dos estudos foi registrada em bancos de dados de registros de ensaios clínicos.^31-34,38,44,46^ Dois estudos foram realizados nos Estados Unidos da América,^33^ enquanto os demais foram realizados na Europa.

Com exceção do estudo MyStromalCell,^34^ que incluiu pacientes com cardiopatia isquêmica e função ventricular esquerda preservada, os demais estudos incluíram pacientes com insuficiência cardíaca associada.

Os estudos Athena I e Athena II,^33^ conduzidos pelo mesmo grupo de pesquisadores, tiveram desenhos semelhantes, exceto pela dose de ADSC: 0,4 × 10^6^ células/kg de peso em Athena I e 0,8 × 10^6^ células/kg de peso em Atenas II. Como o estudo Athena II incluiu apenas 3 pacientes e foi semelhante ao Athena I, os pesquisadores combinaram os dados de ambos ensaios e os publicaram em um único relatório.^33^

Um total de 376 participantes foram incluídos nos estudos, sendo 258 pacientes que receberam transplante de ADSC e 118 pacientes que receberam tratamento médico otimizado com ou sem adição de placebo. Em 3 estudos, o transplante de células-tronco foi combinado com outro tratamento, a saber, cirurgia de revascularização miocárdica,^31^ intervenção coronária percutânea^32^ e revascularização miocárdica por laser.^42^ Apenas um estudo utilizou infusão intracoronária para transplante de ADSC,^32^ enquanto os demais ensaios utilizaram a via intramiocárdica, principalmente por injeção, mas também através de uma placa de gordura em um único ensaio.^31^

A população do estudo foi composta majoritariamente por participantes do sexo masculino, com sobrepeso ou obesidade (índice de massa corporal médio entre 27,5 e 30,8 kg/m^2^), com idade média entre 55 e 67 anos e FEVE média variando de 28,8%^39^ a 54%.^36^ Na maioria dos estudos, os participantes tinham histórico de intervenção coronária percutânea ou cirurgia de revascularização miocárdica prévia. As características dos estudos incluídos e de seus participantes são apresentadas nas Tabela 1 e 2, respectivamente.

**Tabela 1:** – Características dos estudos incluídos

	País	Desenho	N	Tratamento adjuvante	Fonte das células	Dosagem celular	Via de administração	Acompanhamento (meses)
AdiFLAP^31^	Espanha	ECR	I: 5; C: 5	CRM	Autóloga; gordura pericárdica	Desconhecida (enxerto adiposo)	Miocárdica (enxerto adiposo colado)	12
APOLLO^32^	Dinamarca, Espanha e Holanda	ECR	I: 10; C: 4	ICP	Autóloga; gordura periumbilical	20 × 10^6^ células	Intracoronária	6
Athena I^33^	Estados Unidos	ECR	I: 14; C: 14	Nenhum	Autóloga; gordura subcutânea	0,4 × 10^6^ células/kg (máx 40 × 10^6^ células)	Intramiocárdica	12
Athena II^33^	Estados Unidos	ECR	I: 3; C: 0	Nenhum	Autóloga; gordura subcutânea	0,8 × 10^6^ células/kg (máx 80 × 10^6^ células)	Intramiocárdica	12
MyStromalCell^34^	Dinamarca	ECR	I: 41; C: 20	Nenhum	Autóloga; gordura subcutânea abdominal	72 ± 45 × 10^6^ (quantidade total de células alcançada após protocolo de cultura)	Intramiocárdica	36
PRECISE^38^	Dinamarca, Espanha e Holanda	ECR	I: 21; C: 6	Nenhum	Autóloga; gordura subcutânea	0,4 × 10^6^ células/kg (grupo de dosagem baixa); 0,8 × 10^6^ células/kg (grupo de dosagem média)	Intramiocárdica	36
Kastrup et al.^39^	Dinamarca	Não comparativo, de série de casos	I: 10	Nenhum	Alogênica; gordura subcutânea abdominal	100 × 10^6^ células	Intramiocárdica	6
Konstanty- Kalandyk et al.^41-43^	Polônia	Não comparativo, de série de casos	I: 15	Revascularização a laser	Autóloga; gordura subcutânea abdominal	40 × 10^6^	Intramiocárdica	12
DANISH^46^	Dinamarca	ECR	I: 24; C: 27	Nenhum	Alogênica; gordura subcutânea abdominal	100 × 10^6^ células	Intramiocárdica	12
SCIENCE^44^	Dinamarca, Alemanha, Holanda, Áustria, Eslovénia e Polônia	ECR	I: 90; C: 43	Nenhum	Alogênica; gordura subcutânea abdominal	100 × 10^6^ células	Intramiocárdica	12

C: controle; CRM: cirurgia de revascularização miocárdica; ECR: ensaio clínico randomizado; I: intervenção; ICP: intervenção coronária percutânea.

**Tabela 2:** – Características dos participantes

Estudo	Grupo	n	Idade (anos)	Sexo masculino	IMC (kg/m^2^)	Tabagismo	DM	HTN	IM	CRM	ICP	FEVE (%)
AdiFLAP^31^	I	5	63,8 ± 13	5 (100)	ND	5/5 (100)	1/5 (20)	4 (80)	5 (100)	ND	ND	41 ± 18
	C	4	60,3 ± 6	4 (100)	ND	4/4 (100)	2/4 (50)	2 (50)	4 (100)	ND	ND	42 ± 15
APOLLO^32^	I	9	61 ± 2,1	7 (78)	27,5 ± 3	6/9 (66,7)	ND	6 (66,7)	9 (100)	0	9/9 (100)	46,1 ± 2,5
	C	4	55 ± 7,5	4 (100)	27,6 ± 3,3	2/4 (50)	ND	2 (50)	4 (100)	0	4/4 (100)	43,5 ± 3,3
Athena I e II^33^	I	17	64,1 ± 8,2	16 (94,1)	ND	11 (64,7)	8 (47,1)	15 (88,2)	14 (82,4)	13 (76,5)	12 (70,6)	31,1 ± 8,7
	C	14	65,7 ± 7,3	13 (92,9)	ND	10 (71,4)	9 (62,3)	13 (92,9)	14 (100)	10 (71,4)	12 (85,7)	31,8 ± 7,7
MyStromalCell^34^	I	40	65,5 ± 9,7	35 (87,5)	30,0 ± 4,1	31 (77,5)	16 (40)	33 (82,5)	26 (65)	33 (82,5)	28 (70)	52 ± 8
	C	20	65,3 ± 8,7	20 (100)	30,0 ± 4,8	19 (95)	6 (30)	12 (60)	10 (50)	20 (100)	15 (75)	54 ± 8
PRECISE^38^	I	21	65,8 ± 6,3	17 (81)	29,4 ± 4,6	15 (71,4)	8 (38,1)	17 (81)	21 (100)	9 (42,9)	19 (90,5)	36,7 ± 7,5
	C	6	55,7 ± 6,1	4 (66,7)	30,8 ± 4,3	4 (66,7)	3 (50)	5 (83,3)	5 (83,3)	1 (16,7)	5 (83,3)	34,2 ± 9,5
Kastrup et al.^39^	I	10	62,5 ± 6,6	7 (70)	30,2 ± 6,7	6 (60)	3 (30)	5 (50)	10 (100)	4 (40)	7 (70)	28,8 ± 4,1
Konstanty-Kalandyk et al.^41-43^	I	15	65 ± 6,2	12 (80)	29,6 ± 5,6	ND	5 (33)	14 (93)	12 (80)	3 (20)	5 (33)	36,7 ± 13,2
DANISH^46^	I	54	67,0 ± 9,0	44 (81,5)	28,8 ± 5,1	44 (81,4)	14 (25,9)	35 (64,8)	46 (85,2)	31 (57,4)	32 (59,3)	34,2 ± 7,9
	C	27	66,6 ± 8,1	24 (88,9)	26,9 ± 4,3	19 (70,4)	8 (26,6)	15 (55,6)	27 (100,0)	11 (40,7)	21 (77,8)	31,4 ± 7,2
SCIENCE^44^	I	90	66,4 ± 8,1	84 (93,3)	28,5 ± 4,6	75 (83,3)	38 (42,2)	72 (80,0)	69 (76,7)	44 (48,9)	68 (75,6)	31,6 ± 7,2
	C	43	64,0 ± 8,8	38 (88,4)	29,9 ± 3,8	34 (79,1)	17 (39,5)	29 (67,4)	39 (90,7)	15 (34,9)	34 (79,1)	32,0 ± 8,9

Dados apresentados como média ± desvio padrão ou frequência (%). C: controle; CRM: cirurgia de revascularização miocárdica; DM: diabetes mellitus; FEVE: fração de ejeção do ventrículo esquerdo; HTN: hipertensão; I: intervenção; ICP: intervenção coronária percutânea; IM: infarto do miocárdio; IMC: índice de massa corporal; ND: não disponível.

### Risco de viés dos estudos incluídos

Entre os estudos randomizados, apenas um descreveu como foi gerada a lista de randomização.^38^ O cegamento dos pacientes e pesquisadores foi implementado na maioria dos estudos,^32-34,38,44,46^ e os avaliadores do desfecho foram cegados em todos eles. Todos os estudos apresentaram baixo risco de viés em termos de dados incompletos sobre desfechos e relatos seletivos de desfechos.

Entre os estudos não comparativos, Kastrup et al.^39^definiram claramente os objetivos, coletaram dados prospectivamente, consideraram desfechos adequados aos objetivos do estudo, utilizaram tempo de seguimento adequado e tiveram menos de 5% de perdas de seguimento. Porém, o estudo não teve os desfechos avaliados por avaliador independente e não calculou o tamanho amostral prospectivamente. Konstanty-Kalandyk et al.^41-43^ relataram adequadamente todos os domínios, exceto o cálculo do tamanho amostral.

Avaliações detalhadas do risco de viés para os estudos randomizados e não comparativos estão descritas nas Tabelas 3 e 4, respectivamente.

**Tabela 3:** – Avaliação do risco de viés nos ensaios clínicos randomizados

	Geração de sequência aleatória	Ocultação de alocação	Cegamento dos participantes	Cegamento dos investigadores	Cegamentos dos avaliadores de desfecho	Dados incompletos de desfecho	Relato seletivo dos desfechos
AdiFLAP^31^	Provavelmente baixa	Provavelmente baixa	Provavelmente baixa	Provavelmente baixa	Definitivamente baixa	Definitivamente baixa	Definitivamente baixa
APOLLO^32^	Provavelmente baixa	Provavelmente baixa	Definitivamente baixa	Definitivamente baixa	Provavelmente baixa	Definitivamente baixa	Definitivamente baixa
Athena I^33^	Provavelmente baixa	Provavelmente baixa	Definitivamente baixa	Definitivamente baixa	Definitivamente baixa	Definitivamente baixa	Definitivamente baixa
Athena II^33^	Provavelmente baixa	Provavelmente baixa	Definitivamente baixa	Definitivamente baixa	Definitivamente baixa	Definitivamente baixa	Definitivamente baixa
MyStromalCell^34^	Provavelmente baixa	Provavelmente baixa	Definitivamente baixa	Definitivamente baixa	Provavelmente baixa	Definitivamente baixa	Definitivamente baixa
PRECISE^38^	Definitivamente baixa	Provavelmente baixa	Definitivamente baixa	Definitivamente baixa	Definitivamente baixa	Definitivamente baixa	Definitivamente baixa
DANISH^46^	Definitivamente baixa	Definitivamente baixa	Definitivamente baixa	Provavelmente baixa	Definitivamente baixa	Definitivamente baixa	Definitivamente baixa
SCIENCE^44^	Definitivamente baixa	Definitivamente baixa	Definitivamente baixa	Provavelmente baixa	Definitivamente baixa	Definitivamente baixa	Definitivamente baixa

**Tabela 4:** – Avaliação do risco de viés nos estudos não comparativos

	Objetivos claramente definidos	Inclusão de participantes consecutivos	Coleta prospectiva de dados	Desfecho adequado ao objetivo do estudo	Avaliação imparcial dos desfechos	Período de acompanhamento adequado	Perda de acompanhamento menor que 5%	Cálculo prospectivo do tamanho amostral
Kastrup et al.^39^	Adequadamente relatado	Não relatado	Adequadamente relatado	Adequadamente relatado	Não relatado	Adequadamente relatado	Adequadamente relatado	Não relatado
Konstanty-Kalandyk et al.^41-43^	Adequadamente relatado	Adequadamente relatado	Adequadamente relatado	Adequadamente relatado	Adequadamente relatado	Adequadamente relatado	Inadequadamente relatado	Inadequadamente relatado

### Desfechos

#### Segurança

Os eventos adversos foram pouco frequentes e, quando presentes, geralmente relacionados à doença subjacente.

Durante os estudos Athena I e II, 3 pacientes sofreram possíveis ataques isquêmicos transitórios ou acidentes vasculares cerebrais após injeção intramiocárdica: 2 pacientes no grupo experimental e 1 no grupo controle. O comitê independente de monitoramento de eventos recomendou a suspensão temporária do estudo, que foi continuado com uma alteração do protocolo padronizando o uso de antiplaquetários, regimes de anticoagulação pré-operatória, heparina intraprocedimento e exclusão de pacientes com fibrilação atrial.^33^

No estudo MyStromalCell, o grupo controle teve maior necessidade de internação hospitalar por piora da angina em comparação aos pacientes tratados com ADSC (60% versus 35%; p = 0,028).^35^ Não houve diferenças entre os grupos nos demais desfechos de segurança. Os desfechos de segurança relatados nos estudos incluídos são apresentados na Tabela 5.

**Tabela 5:** – Desfechos de segurança

Estudo	Desfecho	Intervenção N eventos / N participantes (%)	Controle N eventos / N participantes (%)
AdiFLAP^31^	Eventos adversos totais*	3 / 5 (60%)	2 / 4 (50%)
	Morte	1 / 5 (20%)	0
	Readmissão hospitalar	1 / 5 (20%)	1 / 4 (25%)
APOLLO^32^	Eventos adversos graves*	2 / 4 (50%)	3 / 9 (33%)
Athena I e II^33^	Eventos adversos graves	9 / 17 (52,9%)	9 / 14 (64,3%)
	ECAM	6 / 17 (35,3%)	3 / 14 (21,4%)
MyStromalCell^34^	Óbito	4 / 40 (10%)	0
	Infarto do miocárdio	8 / 40 (20%)	5 / 20 (25%)
PRECISE^38^	Morte cardíaca*	1 / 21 (4,8%)	1 (16,7%)
	Infarto do miocárdio*	0	1 (16,7%)
Kastrup et al.^39^	Óbito*	1 / 10 (10%)	-
	Hospitalização*	1 / 10 (10%)	-
Konstanty-Kalandyk et al.^41-43^	Óbito	0	-
	Eventos adversos	0	-
DANISH^46^	Óbito	3 / 54 (5,6%)	0
	Hospitalização por infarto do miocárdio	2 / 54 (3,7%)	1 / 27 (3,7%)
	Hospitalização por piora da insuficiência cardíaca	5 / 54 (9,3%)	2 / 27 (7,4%)
SCIENCE^44^	Óbito	3 / 90 (3,3%)	2 / 43 (4,7%)
	Hospitalização por infarto do miocárdio	4 / 90 (4,4%)	1 / 43 (2,3%)
	Hospitalização por piora da insuficiência cardíaca	14 / 90 (15,5%)	7 / 43 (16,3%)

Não houve diferenças estatisticamente significativas entre os grupos em nenhum desses desfechos de segurança. Todos os estudos adotaram significância estatística de 5%. *Desfechos primários do estudo.

#### Eficácia

No ensaio adiFLAP, não foram observadas diferenças significativas entre os grupos em termos de desfechos relacionados à viabilidade miocárdica e à função ventricular esquerda.^31^

No estudo APOLLO, os pacientes que receberam transplante de células-tronco apresentaram redução significativa no defeito perfusional avaliado pela cintilografia (−6%; p = 0,004), enquanto o defeito perfusional no grupo controle permaneceu inalterado (+1,8%; p = não significativo [NS]).^32^ Também houve redução na área de infarto do ventrículo esquerdo de 31,6% para 15,3% (p = 0,002) nos pacientes tratados com células-tronco, enquanto a área média de infarto não se alterou no grupo controle. A função ventricular esquerda permaneceu a mesma no grupo intervenção (+4%; p = NS) e no grupo controle (−1,7%; p = NS).

Os pacientes tratados com células-tronco nos estudos Athena I e II não mostraram diferenças na média do VO_2_ máximo aos 6 meses entre os grupos (+54,9 mL/min; intervalo de confiança de 95% −109 a 219; p = 0,495).^33^ O defeito perfusional do ventrículo esquerdo durante o esforço não apresentou alteração estatisticamente significativa em comparação aos controles (−2,3% vs. 1,2%; p = 0,074). Aos 12 meses, 57% e 67% dos pacientes tratados com células-tronco apresentaram melhora nas classificações da NYHA e CCS, respectivamente, em comparação com 15% e 27% no grupo controle (valor p não relatado). Entretanto, não houve diferenças nos parâmetros relacionados à função ventricular esquerda.

No estudo MyStromalCell,^35^ também foi observada melhora nas classificações NYHA e CCS em relação aos escores médios basais nos pacientes designados para tratamento experimental (p = 0,007 e 0,002, respectivamente), enquanto os pacientes designados para o grupo controle não apresentaram melhora em 36 meses de acompanhamento. O tempo total de exercício e trabalho durante o teste de esforço permaneceu inalterado ao longo do tempo no grupo experimental (p = 0,052 e 0,123, respectivamente), enquanto uma redução significativa foi observada no grupo controle (p = 0,001 e 0,019, respectivamente). Ambos os grupos experimentaram uma redução nos METs em comparação com os valores médios basais durante os 3 anos de acompanhamento, mas não houve diferença entre os grupos. Não foi observada diferença entre os grupos nos parâmetros relacionados à perfusão miocárdica e à função ventricular esquerda.^37^

No estudo PRECISE,^38^ não houve variações significativas nos escores das diferenças estresse-repouso entre os grupos. Entretanto, houve redução no grupo experimental aos 6 meses em relação aos valores basais (de 9,3 para 5,8; p = 0,02), enquanto os valores permaneceram inalterados no grupo controle (de 12,8 para 9,0; p = 0,1). Essas diferenças se mantiveram aos 18 meses (de 8,2 para 5,1; p = 0,03 versus 12,8 para 7,2; p = 0,05, respectivamente). Houve um aumento estatisticamente significativo no índice visual de motilidade parietal aos 6 meses em pacientes tratados com células-tronco (de 25,2 para 27,6; p = 0,03), mas não houve diferenças no grupo controle (de 35,3 para 34,0; p = 0,5). Aos 6 meses, o grupo controle apresentou aumento na área de infarto (p = 0,01), enquanto a área média de infarto permaneceu inalterada no grupo experimental. Os pacientes do grupo controle apresentaram piora dos METs e do VO_2_ máximo (p = 0,001 em ambas as comparações) após 18 meses em relação ao valor basal, enquanto os valores médios permaneceram estáveis no grupo de pacientes que receberam o tratamento experimental (p = 0,1 e 0,8, respectivamente). Não foram observadas alterações significativas na FEVE ou nos volumes do ventrículo esquerdo ao longo do tempo ou entre os grupos (valores não disponíveis).

No estudo de Kastrup et al.,^39^ houve aumento da distância percorrida no teste de caminhada de 6 minutos de 460 m para 495 m em 6 meses de acompanhamento, mas não houve diferenças nos demais estágios avaliados. Konstanty-Kalandyk et al. relataram uma melhora significativa no volume sistólico de 83,1 mL (desvio padrão 8,5) para 93,8 mL (desvio padrão 13,8), conforme avaliado por RM, 1 ano após a intervenção (p = 0,025).^41-43^

Os ensaios DANISH e SCIENCE não mostraram qualquer benefício da intervenção ao compará-la ao grupo placebo nos desfechos primários (alteração no volume sistólico final do ventrículo esquerdo) ou nos desfechos secundários.^44,46^ As únicas indicações de benefício foram um aumento na qualidade de vida medida pelo Questionário de Cardiomiopatia de Kansas City no grupo ADSC no acompanhamento quando comparado ao valor basal (pontuação média 64 ± 3 versus 72 ± 3; p = 0,011) no estudo DANISH, e um pequeno aumento na FEVE do o início até 6 meses de acompanhamento (31,6 ± 7,2 versus 32,8 ± 7,5; p = 0,044) no braço de intervenção do ensaio SCIENCE.

Os desfechos de eficácia relatados nos estudos são apresentados na Tabela 6.

**Tabela 6:** – Desfechos de segurança

	Desfechos	Grupo de intervenção Linha de base versus pós-acompanhamento	Intervenção versus controle	Acompanhamento (meses)
AdiFLAP^31^	Massa necrótica, razão de necrose (RM), FEVE, VSF VE, VDF VE, VS, débito cardíaco (RM)	ND	NS	6-12
APOLLO^32^	Defeito perfusional, % (cintilografia)	16,9 ± 2,1 versus 10,9 ± 2,4; p = 0,004	NS	6
	Área infartada do VE, % (RM)	31,6 ± 5,3 versus 15,3 ± 2,6; p = 0,002	NS	6
	FEVE, % (cintilografia)	NS	NS	6
Athena I e II^33^	Classe NYHA, classe CCS; VO_2_ máx; FEVE, VSF VE, VDF VE (eco); defeito perfusional ao estresse (SPECT)	NS	NS	12
	MLHFQ	ND	–21,6 ± 13,9 versus, –5,5 ± 23,8; p = 0,038	12
	Short Form 36	ND	p < 0,05	12
MyStromalCell^34^	CCS	2,5 ± 0,9 versus 1,8 ± 1,2; p = 0,002	ND	36
	NYHA	2,4 ± 0,6 versus 2,2 ± 0,8; p = 0,007	ND	36
	METs	4,2 ± 0,3 versus 4,0 ± 0,4; p = 0,027	NS	36
	Múltiplos parâmetros de perfusão miocárdica (RM). VDF VE, VSF VE, VS, FEVE, massa miocárdica, massa do tecido fibrótico, tempo de tolerância ao exercício*; desempenho	NS	NS	6-36
PRECISE^38^	METs, massa da área infartada do VE, gramas e % (RM)	NS	ND	6-18
	VO_2_ máx, mL/kg/min	NS	0,3 ± 3,7 versus –4,1 ± 1,5; p = 0,01	18
	Escore somado da diferença estresse-repouso (cintilografia)	9,3 ± 7,0 versus 5,1 ± 3,7; p = 0,02	ND	18
	Escore somado visual da motilidade da parede (RM)	25,2 ± 11,5 versus 27,6 ± 10,8; p = 0,03	ND	6
	Índice de motilidade da parede (RM)	2,1 ± 0,6 versus1,7 ± 0,9; p = 0,04	ND	6
	Massa total do VE, gramas (RM)	128,1 ± 26 versus 149,5 ± 32,4; p < 0,001	ND	6
Kastrup et al.^39^	Distância, metros (TC6)	460 versus 495; p < 0,0001	ND	6
	NYHA, CCS, qualidade de vida (KCCQ); VSF VE, VDF VE, FEVE (eco)	NS	ND	6
Konstanty-Kalandyk et al.^41-43^	CCS, uso de nitratos; FEVE, IVS (RM), FEVE (eco); FEVE, VDF, VSF, débito cardíaco, massa miocárdica, índice cardíaco, taxa de pico de ejeção, taxa de pico de enchimento (RM)	NS	ND	6-12
	VS, mL (MRI)	83,1 ± 8,5 versus 93,8 ± 13,8; p = 0,025	ND	12
	IVS, mL/m^2^ (RM)	43,3 ± 7,6 versus 48,7 ± 9,1; p = 0,019	ND	12
DANISH^46^	VSF VE*, VDF VE, FEVE (eco)	NS	NS	6
	TC6; NYHA	NS	NS	12
	Qualidade de vida (KCCQ)	64 ± 3 versus 72 ± 3; p = 0,011	NS	12
SCIENCE^44^	VSF VE*, VDF VE (eco)	NS	NS	6
	FEVE (eco)	31,6 ± 7,2 versus 32,8 ± 7,5; p = 0,044	NS	6
	TC6, NYHA	NS	NS	12

CCS: Canadian Cardiovascular Society; eco: ecocardiografia; FEVE: fração de ejeção do ventrículo esquerdo; IVS: índice de volume sistólico; KCCQ: Questionário de Cardiomiopatia de Kansas City; MET: equivalente metabólico; ND: não disponível; NS: não significativo; NYHA: New York Heart Association; RM: ressonância magnética; VDF: volume diastólico final; VE: ventrículo esquerdo; VS: volume sistólico; VSF: volume sistólico final; MLHFQ: Minnesota Living with Heart Failure Questionnaire; TC6: teste de caminhada de 6 minutos; VO2 máx: consumo máximo de oxigênio. Os dados são apresentados como média (desvio padrão), mediana (intervalo interquartil) e N quando apropriado. As diferenças entre grupos são relatadas como diferenças absolutas ou relativas. Valores de p para testes estatísticos apropriados relatados nos ensaios originais. Todos os estudos adotaram significância estatística de 5%. *Desfecho primário do estudo.

## Discussão

A presente revisão sistemática visou descrever a segurança e eficácia da terapia com ADSC em estudos clínicos envolvendo pacientes com cardiopatia isquêmica. Foram selecionados 10 estudos com base em critérios de inclusão pré-definidos, incluindo 8 ensaios controlados randomizados e 2 estudos não controlados. A população de pacientes consistiu de indivíduos com cardiopatia isquêmica, com ou sem disfunção ventricular esquerda, uma consideração importante na avaliação de desfechos de eficácia. A maioria dos estudos concentrou-se principalmente na viabilidade e segurança da terapia celular, e as reações adversas graves foram raras. Os desfechos de segurança não mostraram diferenças estatisticamente significativas entre os grupos de tratamento e controle, não indicando qualquer dano da terapia.

Diferentemente dos estudos em animais, os estudos clínicos baseiam-se em métodos indiretos para estimar a perfusão tecidual, uma vez que análises mais invasivas ou histopatológicas não são viáveis. Técnicas de imagem não invasivas, como ecocardiografia, cintilografia e RM, são utilizadas para avaliar a perfusão miocárdica em repouso e sob estresse. Contudo, estudos que avaliaram especificamente a cintilografia miocárdica demonstraram uma redução significativa na isquemia induzida por estresse apenas em pacientes tratados com ADSC.^32,38^ A melhora na contratilidade miocárdica pode refletir indiretamente a melhora da perfusão miocárdica, dada a estreita relação fisiológica entre perfusão tecidual e contratilidade.^47^ Embora a função ventricular esquerda global não tenha diferido significativamente entre os grupos experimental e controle nos estudos analisados, um estudo identificou melhora da motilidade parietal em segmentos tratados com ADSC utilizando RM.^38^ Outro aspecto importante na avaliação de pacientes com cardiopatia isquêmica é a quantificação subjetiva (autorrelatada) e objetiva (teste ergométrico) da limitação funcional causada pela isquemia miocárdica. Em pelo menos 3 estudos,^36,38,39^ a capacidade funcional aumentou em pacientes tratados com células adiposas em comparação ao grupo controle, e um estudo^36^ documentou melhorias subjetivas na classe funcional da angina e na insuficiência cardíaca. Adicionalmente, a remodelação da matriz extracelular, particularmente uma diminuição na área de fibrose pós-infarto, foi observada em 2 estudos,^32,38^ consistente com os achados em um modelo suíno.^21^

Os ensaios incluídos relataram principalmente desfechos substitutos como desfechos de eficácia. Esses foram ensaios de fase I ou II com amostras pequenas, limitando o seu poder para avaliar desfechos clínicos relevantes. Embora as diferenças entre os grupos experimentais e de controle não tenham atingido significância estatística para a maioria dos desfechos substitutos, houve tendências que sugeriram benefícios potenciais. De forma encorajadora, foram observadas algumas melhorias no grupo experimental em comparação com a linha de base, fornecendo suporte para pesquisas futuras. Porém, os dois estudos publicados mais recentemente, os ensaios DANISH^46^ e SCIENCE,^44^ embora reafirmem a segurança, tiveram resultados decepcionantes em termos de eficácia. O uso de um produto celular alogênico padronizado pode ter impactado seus resultados.

Múltiplos mecanismos podem estar subjacentes aos benefícios potenciais das ADSC na cardiopatia isquêmica. A liberação de fatores parácrinos, como citocinas pró-angiogênicas ou antiapoptóticas, pode contribuir para melhorar a vascularização e reduzir a formação de cicatrizes. Além disso, uma fração menor de ADSC também pode se diferenciar em cardiomiócitos, mas sua relevância para a regeneração do tecido miocárdico nunca foi demonstrada.^48^

Outros tipos de células-tronco mesenquimais foram investigados em pacientes com cardiopatia isquêmica. Um recente ensaio clínico randomizado de fase III avaliou o uso de células precursoras mesenquimais derivadas da medula óssea em pacientes com insuficiência cardíaca avançada, predominantemente de origem isquêmica. Embora o ensaio não tenha atingido seus objetivos primários e secundários, análises post-hoc demonstraram benefícios potenciais em certos subgrupos, como pacientes com proteína C reativa de alta sensibilidade elevada.^49^Outro estudo mostrou melhorias sustentadas, ao longo de 12 meses, na isquemia miocárdica regional e reserva de fluxo coronariano associadas ao transplante de células de medula óssea em pacientes com isquemia crônica.^50^ Ensaios prévios demonstraram a segurança e os benefícios potenciais dessas terapias celulares.^51-53^ Células-tronco mesenquimais derivadas do cordão umbilical e de geleia de Wharton também mostraram resultados promissores em pacientes com cardiopatia isquêmica.^54,55^ Embora nenhum estudo clínico tenha comparado diretamente diferentes tipos de células-tronco mesenquimais, elas exibiram perfis de segurança e benefícios semelhantes. Fatores como o custo e os desafios associados à colheita e à expansão dessas células podem influenciar a escolha do tipo de célula mais adequada para o tratamento.

Do ponto de vista clínico, existe uma grande necessidade de novas terapias em pacientes com cardiopatia isquêmica. Apesar do progresso nas técnicas cirúrgicas e nas tecnologias de intervenção coronária percutânea, há um número relevante de pacientes com angina que são candidatos subótimos para revascularização ou nos quais a revascularização não é viável.^22^ Talvez a maior demanda por novas terapias seja para pacientes que desenvolvem insuficiência cardíaca com fração de ejeção reduzida após um evento isquêmico. Esses pacientes têm pior prognóstico quando comparados àqueles com insuficiência cardíaca de outras etiologias e, muitas vezes, evoluem para doença avançada, com sintomas refratários apesar da terapia ideal.^23^ Nesses casos, as opções terapêuticas estão limitadas a dispositivos de assistência ventricular esquerda, que são caros e não estão disponíveis em muitos países ou a transplante cardíaco, que depende da disponibilidade de órgãos e possui múltiplos pré-requisitos para pacientes candidatos. Esse cenário é refletido pelos ensaios sobre ADSC, que incluíram principalmente pacientes sintomáticos com fração de ejeção reduzida. No entanto, esse subgrupo de pacientes apresenta frequentemente doença miocárdica crônica com alto remodelamento e grandes áreas fibróticas; talvez eventuais melhorias na vascularização ou reparo com ADSC possam ocorrer demasiado tarde na história natural da doença. É possível que o ponto ideal esteja mais próximo do evento isquêmico agudo, onde há maior potencial para reduzir a formação de cicatrizes e prevenir a remodelação.

Uma das principais limitações na área é a falta de padronização de preparações celulares, métodos de entrega (por exemplo, infusão intracoronária versus injeção intramiocárdica) e eficácia dos desfechos (por exemplo, FEVE, redução no tamanho do infarto, aumento da perfusão miocárdica ou tolerância ao exercício), o que representa um grande desafio na avaliação da segurança e eficácia das terapias baseadas em células em ensaios clínicos. O estabelecimento de protocolos padronizados para manuseio e entrega de células agilizará o processo translacional e facilitará ensaios clínicos maiores para avaliar essa estratégia terapêutica promissora. Outra limitação do presente estudo é a falta de metanálise ou síntese quantitativa de dados. Contudo, considerando a grande heterogeneidade dos estudos, presumimos que uma abordagem quantitativa para a síntese dos dados poderia levar a conclusões equivocadas e, portanto, optamos por uma síntese narrativa.

## Conclusões

Com base em pequenos estudos de pacientes com cardiopatia isquêmica, a injeção de ADSC parece ser segura e mostrou alguns efeitos benéficos preliminares. Investigações adicionais são necessárias com o objetivo de diminuir as respostas inflamatórias e fibróticas e melhorar a função da microcirculação cardíaca nesses pacientes. Embora a intervenção pareça viável, segura e promissora, são necessários ensaios clínicos maiores para avaliar a eficácia das ADSC em pacientes com cardiopatia isquêmica.
