# Supplementary material for: Segurança e Eficácia da Terapia com Células-tronco Mesenquimais Derivadas do Tecido Adiposo para Cardiopatia Isquêmica: Revisão Sistemática
Source: Arq Bras Cardiol. 2024 Sep 6;121(9):e20230830. [Article in Portuguese] doi: 10.36660/abc.20230830 (PMC11495568; doi:10.36660/abc.20230830)
Supplement: Translation

# Translation: Safety and Efficacy of Adipose-Derived Mesenchymal Stem Cell Therapy for Ischemic Heart Disease: A Systematic Review

**DOI:** 10.36660/abc.20230830i

**Authors:** Fernando Rabioglio Giugni, Melina de Oliveira Valdo Giugni, Henrique Trombini Pinesi, Fabio Cetinic Habrum, Lígia Nasi Laranjeira, Erica Regina Ribeiro Sady, Erica Aranha Suzumura, Luis Henrique Wolff Gowdak, José Eduardo Krieger

**Affiliations:** 1 USA The University of Texas Southwestern Medical Center, Dallas, Texas – USA; 2 Brazil Instituto do Coração InCor, Hospital das Clinicas HCFMUSP, Faculdade de Medicina, Universidade de São Paulo, São Paulo, SP – Brazil; 3 USA Baylor University Medical Center at Dallas, Dallas, Texas – USA; 4 Brazil Hospital do Coração (HCor), São Paulo, SP – Brazil; 5 Brazil Departmento de Medicina Preventiva, Faculdade de Medicina FMUSP, Universidade de São Paulo, São Paulo, SP – Brazil

**Keywords:** Mesenchymal Stem Cells, Regenerative Medicine, Myocardial Ischemia, Cell- and Tissue-Based Therapy

## Abstract

**Background:**

Cell therapy using adipose-derived mesenchymal stem cells (ADSCs) shows great potential as a treatment for cardiovascular diseases.

**Objective:**

We conducted a systematic review to describe the safety and efficacy of ADSCs in ischemic heart disease.

**Methods:**

We searched PubMed/MEDLINE, EMBASE, Web of Science, CENTRAL, and LILACS (from inception to March 2024) for clinical studies involving ADSCs in patients with ischemic heart disease. We excluded studies involving patients with other types of heart disease, studies using mesenchymal stem cells derived from other tissues, as well as ongoing studies. Two independent reviewers screened the retrieved citations, extracted relevant data, and assessed the risk of bias in the included trials, using the Cochrane Collaboration criteria modified by McMaster University and Methodological Index for Non-Randomized Studies (MINORS). We used a narrative synthesis to present the results.

**Results:**

Ten studies (comprising 29 publications) met our inclusion criteria, including 8 randomized controlled trials and 2 uncontrolled trials. No severe adverse events associated with ADSC therapy were reported. While most efficacy endpoints did not reach statistical significance, there were reports of improved ischemic area, functional capacity, symptoms, and contractility in patients treated with ADSCs.

**Conclusions:**

The findings from our review suggest that ADSC therapy is generally safe for patients with ischemic heart disease. However, further investigation is warranted to confirm its efficacy, particularly with larger clinical trials and in specific conditions where improvements in microcirculation may have a notable impact on clinical outcomes.

## Introduction

Over the past two decades, stem cell therapy has emerged as a promising approach to treating various conditions that have limited responses or do not respond to conventional therapies. Initially, the focus was on the regenerative capacity of stem cells, their ability to self-renew and differentiate into different cell types.^1-3^ However, recent evidence suggests that the therapeutic effects of stem cell therapy are primarily mediated through paracrine factors, which modulate the body’s natural response to injury, both acute and chronic.^4,5^

Extensive *in vitro* studies have characterized various types of progenitor cells, and animal models have shown promising results in evaluating the effectiveness of stem cell therapy for different conditions. This progress paved the way for the first clinical trials involving the use of autologous or allogenic adult stem cells. Several types of adult stem cells have been investigated, including skeletal myoblasts, bone marrow-derived cells, cardiac stem cells, blood-derived endothelial progenitor cells, and adipose tissue-derived stem cells (ADSCs).^2,6-9^

Adipose tissue, which originates from the embryonic mesenchyme, provides a readily accessible source of stromal cells.^10^ ADSCs can be isolated from human liposuction residues after treatment with collagenase and centrifugation. Similar to other mesenchymal stem cells, ADSCs can be induced to differentiate into various cell subtypes *in vitro* by modifying the cell culture medium with specific factors.^11^ Due to their versatile nature, ADSCs have been extensively studied in the field of regenerative medicine, with applications ranging from chronic skin wounds and soft tissue defects to inflammatory bowel diseases, type 1 diabetes mellitus, spinal cord injuries, and stroke.^12-16^

Under specific experimental conditions, ADSCs can also differentiate into cells of the cardiovascular system.^17,18^ Furthermore, ADSCs release paracrine factors that modulate the properties of the tissue microenvironment.^5,19^ These factors promote neovascularization, reduce apoptosis and inflammation, and inhibit fibrosis, thereby enhancing cardiac repair and functional recovery. Preclinical studies have provided substantial evidence supporting the potential of ADSCs for cardiac repair in humans.^7,20,21^ Additionally, ADSCs can be obtained in large quantities and expanded for future therapeutic use, which is advantageous for cell-based therapies.^11^

Patients with advanced coronary artery disease who experience refractory angina or ischemic heart failure represent a significant clinical challenge. Antianginal medications may not adequately alleviate symptoms, and myocardial revascularization procedures may not be feasible due to poor distal arterial flow or diffuse atherosclerotic obstructive disease, among other reasons.^22^ For some individuals with progressive ischemic heart failure, heart transplantation becomes the only viable option to improve survival and quality of life despite optimized pharmacological treatment.^23^ In these situations, treatment with ADSCs could serve as an alternative therapeutic strategy, aiming to enhance neo-angio/vasculogenesis, ameliorate endothelial dysfunction, and reduce inflammation and fibrosis, collectively referred to as cardiac repair. However, the safety and efficacy of ADSC therapy for these conditions are still being investigated.

To gain insight into the current status of ADSC therapy for ischemic heart disease, we conducted a systematic review of clinical studies (Central Figure). Our aim was to identify the existing knowledge gaps and areas that require further investigation to advance this therapeutic approach.

## Methods

This systematic review adhered to the recommended guidelines of the Cochrane Collaboration,^24,25^ and the results were reported following the Preferred Reporting Items for Systematic Reviews and Meta-analyses (PRISMA).^26^

### Data sources and searches

We conducted comprehensive searches in the following electronic databases (from inception to March 2024): PubMed via MEDLINE, EMBASE, Web of Science, Cochrane Library/CENTRAL, and LILACS. No language restrictions were applied, and controlled vocabulary was utilized whenever possible (MeSH term for MEDLINE and CENTRAL, EMTREE for EMBASE, and DeCS for LILACS). To enhance the search strategy, we employed keywords and their synonyms. The complete search strategy is reported in Supplementary Table 1. Additionally, we manually searched the reference lists of the included studies to identify other relevant articles.

### Eligibility criteria

We included studies that met the following criteria: (1) full-text publications with the following research designs: randomized or quasi-randomized trials, comparative observational studies, or non-comparative case series involving at least 10 patients; (2) involving patients with acute or chronic ischemic heart disease; (3) assessing the effects of ADSCs transplantation; (4) reporting at least one of the outcomes of interest. We excluded studies involving patients with other types of heart disease or ischemia in organs other than the heart (e.g., peripheral, brain, renal); studies using mesenchymal stem cells derived from other tissues such as bone marrow, umbilical cord, synovial tissue, or peripheral blood; as well as ongoing studies.

### Study selection

Two reviewers independently screened the titles and abstracts of all retrieved citations. If at least one reviewer considered a citation potentially suitable, the full-text publication was obtained and thoroughly assessed to confirm eligibility. In cases where selected studies were published in multiple journals (multiple publications) or included sub-studies, the data were listed under the primary reference to provide additional information. Studies published solely as conference abstracts were deemed ineligible due to limited information. Disagreements between reviewers were resolved through discussion, consensus, or consultation with a third reviewer.

### Data extraction and risk of bias assessment

Two reviewers independently extracted data from eligible studies using a standardized form and evaluated the risk of bias based on domain-specific criteria. For randomized or quasi-randomized trials, the Cochrane Collaboration criteria^25^ modified by McMaster University^27^ were employed. Observational studies were assessed using the Risk Of Bias In Non-randomized Studies of Interventions (ROBINS-I) tool,^28^ while non-comparative studies were evaluated using the Methodological Index for Non-Randomized Studies (MINORS).^29^ Disagreements among reviewers were resolved through discussion, consensus, or consultation with a third reviewer.

### Outcomes

The efficacy outcomes of interest included myocardial biopsy, collateral neovascularization (coronary angiography), Canadian Cardiovascular Society (CCS) grading of angina, exercise test performance (metabolic equivalents [METs], tolerance time, load [watts]), myocardial perfusion (myocardial scintigraphy, magnetic resonance imaging [MRI], stress echocardiogram, cardiac positron emission tomography [PET]), and myocardial viability (myocardial scintigraphy, MRI, stress echocardiogram, cardiac PET). In patients with pre-intervention myocardial ischemia and heart failure, additional outcomes were New York Heart Association (NYHA) functional classification and left ventricular ejection fraction (LVEF). All effect measures were collected from each outcome.

Safety outcomes were recorded as main adverse events reported in the primary studies.

### Data synthesis

We conducted a narrative synthesis of the results following the guidelines of the European Social Research Council Guidance on the Conduct of Narrative Synthesis in Systematic Reviews^30^ to address our review questions. Findings regarding the characteristics of the included studies, patients, and interventions used, as well as efficacy and safety outcomes, were presented in evidence tables.

## Results

The search strategy yielded 4,285 citations, of which 446 were excluded due to duplication. After screening the titles and abstracts, 3,839 citations were reviewed. Among them, 87 relevant citations were selected for further analysis by reading the full publications. Subsequently, 58 publications were excluded because they did not meet all the eligibility criteria of this systematic review. The reasons for excluding articles after full publication review are illustrated in Figure 1. Finally, 10 studies (comprising 29 publications) evaluating the safety and efficacy of ADSC transplantation for myocardial neo-angio/vasculogenesis in patients with acute or chronic ischemic heart disease were included.^31-46^ The search and selection flowchart of the studies is presented in Figure 1.

**Figure 1:** – Flowchart of search and selection of studies.

### Characteristics of the included studies

The analysis comprised 8 randomized studies^31-33,35,38,44,46^ and 2 uncontrolled studies^39,43^ published between 2012 and 2023. Most studies were registered in clinical trial records databases.^31-34,38,44,46^ Two studies were conducted in the United States of America,^33^ while the others were conducted in Europe.

With the exception of the MyStromalCell study,^34^ which included patients with ischemic heart disease and preserved left ventricular function, the remaining studies included patients with associated heart failure.

The Athena I and Athena II studies,^33^ conducted by the same group of researchers, had similar designs, except for the ADSC dose: 0.4 × 10^6^ cells/kg of weight in Athena I and 0.8 × 10^6^ cells/kg of weight in Athena II. Since the Athena II study included only 3 patients and was similar to Athena I, researchers combined data from both trials and published them as a single report.^33^

A total of 376 participants were included in the studies, with 258 patients receiving ADSC transplantation and 118 patients receiving optimized medical treatment with or without the addition of placebo. In 3 studies, stem cell transplantation was combined with another treatment, namely coronary artery bypass graft,^31^ percutaneous coronary intervention,^32^ and myocardial revascularization by laser.^42^ Only one study utilized intracoronary infusion for ADSC transplantation,^32^ while the other trials used intramyocardial route, mostly by injection, but also through a fat patch in a single trial.^31^

The study population mostly consisted of male participants who were overweight or obese (mean body mass index between 27.5 and 30.8 kg/m^2^), with a mean age between 55 and 67 years, and a mean LVEF ranging from 28.8%^39^ to 54%.^36^ In most studies, participants had a history of previous percutaneous coronary intervention or coronary artery bypass graft. The characteristics of the included studies and their participants are presented in Table 1 and Table 2, respectively.

**Table 1:** – Characteristics of the included studies

	Country	Design	N	Adjunct treatment	Cell source	Cell dosage	Route of administration	Follow-up (months)
AdiFLAP^31^	Spain	RCT	I: 5; C: 5	CABG	Autologous; pericardial fat	Unknown (adipose graft)	Myocardial (adipose patch glued)	12
APOLLO^32^	Denmark, Spain, and Netherlands	RCT	I: 10; C: 4	PCI	Autologous; periumbilical fat	20 × 10^6^ cells	Intracoronary	6
Athena I^33^	United States	RCT	I: 14; C: 14	None	Autologous; subcutaneous fat	0.4 × 10^6^ cells/kg (max 40 × 10^6^ cells)	Intramyocardial	12
Athena II^33^	United States	RCT	I: 3; C: 0	None	Autologous; subcutaneous fat	0.8 × 10^6^ cells/kg (max 80 × 106 cells)	Intramyocardial	12
MyStromalCell^34^	Denmark	RCT	I: 41; C: 20	None	Autologous; abdominal subcutaneous fat	72 ± 45 × 10^6^ (total amount of cells reached after culture protocol)	Intramyocardial	36
PRECISE^38^	Denmark, Spain, and Netherlands	RCT	I: 21; C: 6	None	Autologous; subcutaneous fat	0.4 × 10^6^ cells/kg (low dose group); 0.8 × 10^6^ cells/kg (mid dose group)	Intramyocardial	36
Kastrup et al.^39^	Denmark	Non-comparative case series	I: 10	None	Allogeneic; abdominal subcutaneous fat	100 × 10^6^ cells	Intramyocardial	6
Konstanty- Kalandyk et al.^41-43^	Poland	Non-comparative case series	I: 15	Laser revascularization	Autologous; abdominal subcutaneous fat	40 × 10^6^	Intramyocardial	12
DANISH^46^	Denmark	RCT	I: 24; C: 27	None	Allogeneic; abdominal subcutaneous fat	100 × 10^6^ cells	Intramyocardial	12
SCIENCE^44^	Denmark, Germany, Netherlands, Austria, Slovenia, and Poland	RCT	I: 90; C: 43	None	Allogeneic; abdominal subcutaneous fat	100 × 10^6^ cells	Intramyocardial	12

C: control; CABG: coronary artery bypass graft surgery; I: intervention; PCI: percutaneous coronary intervention; RCT: randomized clinical trial.

**Table 2:** – Participants’ characteristics

Study	Group	n	Age (years)	Male sex	BMI (kg/m^2^)	Smoking	DM	HTN	MI	CABG	PCI	LVEF (%)
AdiFLAP^31^	I	5	63.8 ± 13	5 (100)	ND	5/5 (100)	1/5 (20)	4 (80)	5 (100)	ND	ND	41 ± 18
	C	4	60.3 ± 6	4 (100)	ND	4/4 (100)	2/4 (50)	2 (50)	4 (100)	ND	ND	42 ± 15
APOLLO^32^	I	9	61 ± 2.1	7 (78)	27.5 ± 3	6/9 (66.7)	ND	6 (66.7)	9 (100)	0	9/9 (100)	46.1 ± 2.5
	C	4	55 ± 7.5	4 (100)	27.6 ± 3.3	2/4 (50)	ND	2 (50)	4 (100)	0	4/4 (100)	43.5 ± 3.3
Athena I e II^33^	I	17	64.1 ± 8.2	16 (94.1)	ND	11 (64.7)	8 (47.1)	15 (88.2)	14 (82.4)	13 (76.5)	12 (70.6)	31.1 ± 8.7
	C	14	65.7 ± 7.3	13 (92.9)	ND	10 (71.4)	9 (62.3)	13 (92.9)	14 (100)	10 (71.4)	12 (85.7)	31.8 ± 7.7
MyStromalCell^34^	I	40	65.5 ± 9.7	35 (87.5)	30.0 ± 4.1	31 (77.5)	16 (40)	33 (82.5)	26 (65)	33 (82.5)	28 (70)	52 ± 8
	C	20	65.3 ± 8.7	20 (100)	30.0 ± 4.8	19 (95)	6 (30)	12 (60)	10 (50)	20 (100)	15 (75)	54 ± 8
PRECISE^38^	I	21	65.8 ± 6.3	17 (81)	29.4 ± 4.6	15 (71.4)	8 (38.1)	17 (81)	21 (100)	9 (42.9)	19 (90.5)	36.7 ± 7.5
	C	6	55.7 ± 6.1	4 (66.7)	30.8 ± 4.3	4 (66.7)	3 (50)	5 (83.3)	5 (83.3)	1 (16.7)	5 (83.3)	34.2 ± 9.5
Kastrup et al.^39^	I	10	62.5 ± 6.6	7 (70)	30.2 ± 6.7	6 (60)	3 (30)	5 (50)	10 (100)	4 (40)	7 (70)	28.8 ± 4.1
Konstanty-Kalandyk et al.^41-43^	I	15	65 ± 6.2	12 (80)	29.6 ± 5.6	ND	5 (33)	14 (93)	12 (80)	3 (20)	5 (33)	36.7 ± 13.2
DANISH^46^	I	54	67.0 ± 9.0	44 (81.5)	28.8 ± 5.1	44 (81.4)	14 (25.9)	35 (64.8)	46 (85.2)	31 (57.4)	32 (59.3)	34.2 ± 7.9
	C	27	66.6 ± 8.1	24 (88.9)	26.9 ± 4.3	19 (70.4)	8 (26.6)	15 (55.6)	27 (100.0)	11 (40.7)	21 (77.8)	31.4 ± 7.2
SCIENCE^44^	I	90	66.4 ± 8.1	84 (93.3)	28.5 ± 4.6	75 (83.3)	38 (42.2)	72 (80.0)	69 (76.7)	44 (48.9)	68 (75.6)	31.6 ± 7.2
	C	43	64.0 ± 8.8	38 (88.4)	29.9 ± 3.8	34 (79.1)	17 (39.5)	29 (67.4)	39 (90.7)	15 (34.9)	34 (79.1)	32.0 ± 8.9

Data presented as mean ± standard deviation or frequency (%). BMI: body mass index; C: control; CABG: coronary artery bypass graft surgery; DM: diabetes mellitus; HTN: hypertension; I: intervention; LVEF: left ventricular ejection fraction; MI: myocardial infarction; NA: not available; PCI: percutaneous coronary intervention.

#### Risk of bias of the included studies

Among the randomized studies, only one described how the randomization list was generated.^38^ Blinding of patients and researchers was implemented in most studies,^32-34,38,44,46^ and outcome evaluators were blinded in all of them. All studies exhibited a low risk of bias in terms of incomplete data on outcomes and selective reporting of outcomes.

Among the non-comparative studies, Kastrup et al.^39^clearly defined the objectives, prospectively collected data, considered outcomes suitable to the study objectives, used an appropriate follow-up time, and had less than 5% follow-up losses. However, the study did not have the outcomes assessed by an independent evaluator and did not calculate the sample size prospectively. Konstanty-Kalandyk et al.^41-43^ adequately reported all domains except for sample size calculation.

Detailed assessments of the risk of bias for randomized and non-comparative studies are described in Tables 3 and 4, respectively.

**Table 3:** – Risk of bias assessment in the randomized clinical trials

	Random sequence generation	Allocation concealment	Participant blinded	Investigator blinded	Outcome assessors blinded	Incomplete outcome data	Selective outcome report
AdiFLAP^31^	Probably low	Probably low	Probably low	Probably low	Definitely low	Definitely low	Definitely low
APOLLO^32^	Probably low	Probably low	Definitely low	Definitely low	Probably low	Definitely low	Definitely low
Athena I^33^	Probably low	Probably low	Definitely low	Definitely low	Definitely low	Definitely low	Definitely low
Athena II^33^	Probably low	Probably low	Definitely low	Definitely low	Definitely low	Definitely low	Definitely low
MyStromalCell^34^	Probably low	Probably low	Definitely low	Definitely low	Probably low	Definitely low	Definitely low
PRECISE^38^	Definitely low	Probably low	Definitely low	Definitely low	Definitely low	Definitely low	Definitely low
DANISH^46^	Definitely low	Definitely low	Definitely low	Probably low	Definitely low	Definitely low	Definitely low
SCIENCE^44^	Definitely low	Definitely low	Definitely low	Probably low	Definitely low	Definitely low	Definitely low

**Table 4:** – Risk of bias assessment in the non-comparative studies

	Objectives properly defined	Inclusion of consecutive participants	Prospective data collection	Outcome appropriate for the objective of the study	Unbiased assessment of outcomes	Appropriate follow-up time	Follow-up loss less than 5%	Prospective sample size calculation
Kastrup et al.^39^	Properly reported	Not reported	Properly reported	Properly reported	Not reported	Properly reported	Properly reported	Not reported
Konstanty-Kalandyk et al.^41-43^	Properly reported	Properly reported	Properly reported	Properly reported	Properly reported	Properly reported	Inappropriately reported	Inappropriately reported

## Outcomes

### Safety

Adverse events were infrequent and, when present, usually related to the underlying disease.

During the Athena I and II studies, 3 patients suffered possible transient ischemic attacks or strokes following intramyocardial injection: 2 patients in the experimental group and 1 in the control group. The independent event monitoring committee recommended a temporary suspension of the study, and it was continued with a protocol amendment standardizing the use of antiplatelet drugs, preoperative anticoagulation regimens, intra-procedure heparin, and the exclusion of patients with atrial fibrillation.^33^

In the MyStromalCell study, the control group had a higher need for hospitalization due to worsening of angina compared to patients treated with ADSCs (60% versus 35%; p = 0.028).^35^ There were no differences between the groups in other safety outcomes. The safety outcomes reported in the included studies are presented in Table 5.

**Table 5:** – Safety outcomes

Study	Outcome	Intervention N events / N participants (%)	Control N events / N participants (%)
AdiFLAP^31^	Total adverse events*	3 / 5 (60%)	2 / 4 (50%)
	Death	1 / 5 (20%)	0
	Readmission	1 / 5 (20%)	1 / 4 (25%)
APOLLO^32^	Serious adverse events*	2 / 4 (50%)	3 / 9 (33%)
Athena I e II^33^	Serious adverse events	9 / 17 (52.9%)	9 / 14 (64.3%)
	MACE	6 / 17 (35.3%)	3 / 14 (21.4%)
MyStromalCell^34^	Death	4 / 40 (10%)	0
	Myocardial infarction	8 / 40 (20%)	5 / 20 (25%)
PRECISE^38^	Cardiac death*	1 / 21 (4.8%)	1 (16.7%)
	Myocardial infarction*	0	1 (16.7%)
Kastrup et al.^39^	Death*	1 / 10 (10%)	-
	Hospitalization*	1 / 10 (10%)	-
Konstanty-Kalandyk et al.^41-43^	Death	0	-
	Adverse events	0	-
DANISH^46^	Death	3 / 54 (5.6%)	0
	Hospitalization for myocardial infarction	2 / 54 (3.7%)	1 / 27 (3.7%)
	Hospitalization for worsening heart failure	5 / 54 (9.3%)	2 / 27 (7.4%)
SCIENCE^44^	Death	3 / 90 (3.3%)	2 / 43 (4.7%)
	Hospitalization for myocardial infarction	4 / 90 (4.4%)	1 / 43 (2.3%)
	Hospitalization for worsening heart failure	14 / 90 (15.5%)	7 / 43 (16.3%)

There were no statistically significant differences between groups in any of these safety outcomes. All studies adopted 5% statistical significance. *Primary study endpoints.

### Efficacy

In the adiFLAP Trial, no significant differences were observed between the groups in terms of outcomes related to myocardial viability and left ventricular function.^31^

In the APOLLO study, patients who received stem cell transplantation showed a significant reduction in the perfusion defect evaluated by scintigraphy (−6%; p = 0.004), whereas the perfusion defect in the control group remained unchanged (+1.8%; p = not significant [NS]).^32^ There was also a reduction in the area of left ventricle infarction from 31.6% to 15.3% (p = 0.002) in patients treated with stem cells, while the mean infarction area did not change in the control group. The left ventricular function remained the same in the intervention group (+4%; p = NS) and the control group (−1.7%; p = NS).

Patients treated with stem cells in the Athena I and II studies showed no differences in the mean maximum VO_2_ at 6 months between the groups (+54.9 mL/min; 95% confidence interval −109 to 219; p = 0.495).^33^ The left ventricle perfusion defect during exertion did not exhibit a statistically significant change compared to controls (−2.3% vs. 1.2%; p = 0.074). At 12 months, 57% and 67% of patients treated with stem cells showed improvement in the NYHA and CCS classification, respectively, compared to 15% and 27% in the control group (p value not reported). However, there were no differences in parameters related to left ventricular function.

In the MyStromalCell study,^35^ an improvement in the NYHA and CCS classification was also observed in relation to baseline mean scores in patients assigned to experimental treatment (p = 0.007 and 0.002, respectively), while patients assigned to the control group showed no improvement at 36 months of follow-up. The total exercise time and work during the stress test remained unchanged over time in the experimental group (p = 0.052 and 0.123, respectively), while a significant reduction was observed in the control group (p = 0.001 and 0.019, respectively). Both groups experienced a reduction in METs compared to baseline mean values during the 3 years of follow-up, but there was no difference between the groups. No difference between the groups was observed in parameters related to myocardial perfusion and left ventricular function.^37^

In the PRECISE study,^38^ there were no significant variations in the scores of stress-rest differences between the groups. However, there was a reduction in the experimental group at 6 months compared to baseline values (from 9.3 to 5.8; p = 0.02), whereas the values remained unchanged in the control group (from 12.8 to 9.0; p = 0.1). These differences were maintained at 18 months (from 8.2 to 5.1; p = 0.03 versus from 12.8 to 7.2; p = 0.05, respectively). There was a statistically significant increase in the visual index of parietal motility at 6 months in patients treated with stem cells (from 25.2 to 27.6; p = 0.03), but there were no differences in the control group (from 35.3 to 34.0; p = 0.5). At 6 months, the control group showed an increase in the infarction area (p = 0.01), while the mean area of infarction remained unchanged in the experimental group. Patients in the control group experienced worsening of METs and maximum VO_2_ (p = 0.001 in both comparisons) after 18 months compared to baseline, whereas the mean values remained stable in the group of patients who received the experimental treatment (p = 0.1 and 0.8, respectively). No significant changes were observed in LVEF or left ventricular volumes over time or between the groups (values not available).

In the study conducted by Kastrup et al.^39^ there was an increase in the distance traveled on the 6-minute walk test from 460 m to 495 m in 6 months of follow-up, but there were no differences in the other outcomes evaluated. Konstanty-Kalandyk et al. reported a significant improvement in systolic volume from 83.1 mL (standard deviation 8.5) to 93.8 mL (standard deviation 13.8), as assessed by MRI, 1 year after the intervention (p = 0.025).^41-43^

The DANISH and SCIENCE trials showed no benefit of the intervention when compared to the placebo group on either primary endpoints (change in left ventricle end-systolic volume) or secondary endpoints.^44,46^ The only indications of benefit were an increase in quality of life measured by the Kansas City Cardiomyopathy Questionnaire in the ADSC group at follow-up when compared to baseline (mean score 64 ± 3 versus 72 ± 3; p = 0.011) in the DANISH trial, and a small increase in LVEF from baseline to 6 month follow-up (31.6 ± 7.2 versus 32.8 ± 7.5; p = 0.044) in the intervention arm in the SCIENCE trial.

The efficacy outcomes reported in the studies are presented in Table 6.

**Table 6:** – Efficacy outcomes

	Outcomes	Intervention group Baseline versus after follow-up	Intervention versus control	Follow-up (months)
AdiFLAP^31^	Necrosis mass, necrosis ratio (MRI), LVEF, LV ESV, LV EDV, stroke volume, cardiac output (MRI)	NA	NS	6-12
APOLLO^32^	Perfusion defect, % (scintigraphy)	16.9 ± 2.1 versus 10.9 ± 2.4; p = 0.004	NS	6
	LV infarcted area, % (MRI)	31.6 ± 5.3 versus 15.3 ± 2.6; p = 0.002	NS	6
	LVEF, % (scintigraphy)	NS	NS	6
Athena I e II^33^	NYHA class, CCS class; VO2 max; LVEF, LV ESV, LV EDV (Echo); stress perfusion defect (SPECT)	NS	NS	12
	MLHFQ	NA	–21.6 ± 13.9 versus –5.5 ± 23.8; p = 0.038	12
	Short Form 36	NA	p < 0.05	12
MyStromalCell^34^	CCS	2.5 ± 0.9 versus 1.8 ± 1.2; p = 0.002	NA	36
	NYHA	2.4 ± 0.6 versus 2.2 ± 0.8; p = 0.007	NA	36
	METs	4.2 ± 0.3 versus 4.0 ± 0.4; p = 0.027	NS	36
	Multiple parameters of myocardial perfusion (MRI). LV EDV, LV ESV, stroke volume, LVEF, myocardial mass, fibrotic tissue mass, exercise tolerance time*; performance	NS	NS	6-36
PRECISE^38^	METs, mass infarcted area of LV, grams and % (MRI)	NS	NA	6-18
	VO2 max, mL/kg/min	NS	0.3 ± 3.7 versus –4,1 ± 1,5; p = 0,01	18
	Summed stress-rest difference score (scintigraphy)	9.3 ± 7.0 versus 5.1 ± 3.7; p = 0.02	NA	18
	Visual summed wall motion score (MRI)	25.2 ± 11.5 versus 27.6 ± 10.8; p = 0.03	NA	6
	Wall motion score index (MRI)	2.1 ± 0.6 versus1.7 ± 0.9; p = 0.04	NA	6
	Total LV sass, grams (MRI)	128.1 ± 26 versus 149.5 ± 32.4; p < 0.001	NA	6
Kastrup et al.^39^	Distance covered, meters (6MWT)	460 versus 495; p < 0.0001	NA	6
	NYHA, CCS, quality of life (KCCQ); LV ESV, LV EDV, LVEF (echo)	NS	NA	6
Konstanty-Kalandyk et al.^41-43^	CCS, nitrate use; LVEF, SVI (MRI), LVEF (Echo); LVEF, EDV, ESV, cardiac output, myocardial mass, cardiac index, peak ejection rate index, peak filling rate index (MRI)	NS	NA	6-12
	SV, mL (MRI)	83.1 ± 8.5 versus 93.8 ± 13.8; p = 0.025	NA	12
	SVI, mL/m^2^ (MRI)	43.3 ± 7.6 versus 48.7 ± 9.1; p = 0.019	NA	12
DANISH^46^	LV ESV*, LV EDV, LVEF (echo)	NS	NS	6
	6MWT; NYHA	NS	NS	12
	Quality of life (KCCQ)	64 ± 3 versus 72 ± 3; p = 0.011	NS	12
SCIENCE^44^	LV ESV*, LV EDV (echo)	NS	NS	6
	LVEF (echo)	31.6 ± 7.2 versus 32.8 ± 7.5; p = 0.044	NS	6
	6MWT, NYHA	NS	NS	12

CCS: Canadian Cardiovascular Society; echo: echocardiography; EDV: end-diastolic volume; ESV: end-systolic volume; LV: left ventricular; KCCQ: Kansas City Cardiomyopathy Questionnaire; LVEF: left ventricular ejection fraction; METs: metabolic equivalents; MLHFQ: Minnesota Living with Heart Failure Questionnaire; MRI: magnetic resonance imaging; NA: not available; NS: not significant; NYHA: New York Heart Association; SPECT: single-photon emission computed tomography; SV: systolic volume; SVI: systolic volume index; VO_2_ max: maximal oxygen consumption; 6MWT: 6-minute walk test. Data are shown as mean (standard deviation), median (interquartile range), and n when appropriate. Differences between groups are reported as absolute or relative differences. P value for appropriate statistical tests reported in the original trials. All studies adopted 5% statistical significance. *Primary study endpoint.

## Discussion

This systematic review aimed to describe the safety and effectiveness of ADSC therapy in clinical studies involving patients with ischemic heart disease. Ten studies were selected based on predefined inclusion criteria, including eight randomized controlled trials and two uncontrolled studies. The patient population consisted of individuals with ischemic heart disease, with or without left ventricular dysfunction, an important consideration when assessing efficacy outcomes. Most studies primarily focused on the feasibility and safety of cell therapy, and severe adverse reactions were rare. Safety endpoints showed no statistically significant differences between the treatment and control groups, indicating no harm from the therapy.

Unlike animal studies, clinical studies rely on indirect methods to estimate tissue perfusion since more invasive or histopathological analyses are not feasible. Non-invasive imaging techniques such as echocardiography, scintigraphy, and MRI are used to assess myocardial perfusion at rest and under stress. Nevertheless, studies specifically evaluating myocardial scintigraphy demonstrated a significant reduction in stress-induced ischemia only in patients treated with ADSCs.^32,38^ Improvement in myocardial contractility can indirectly reflect enhanced myocardial perfusion, given the close physiological relationship between tissue perfusion and contractility.^47^ While overall left ventricular function did not significantly differ between the experimental and control groups in the analyzed studies, one study identified improved parietal motility in segments treated with ADSCs using resonance imaging.^38^ Another important aspect in evaluating patients with ischemic heart disease is the subjective (self-reported) and objective (exercise test) quantification of functional limitation caused by myocardial ischemia. In at least three studies,^36,38,39^functional capacity increased in patients treated with adipose cells compared to the control group, and one study^36^ documented subjective improvements in angina functional class and heart failure. Additionally, remodeling of the extracellular matrix, particularly a decrease in post-infarction fibrosis area, was observed in two studies,^32,38^ consistent with findings in a swine model.^21^

The included trials primarily reported surrogate endpoints as efficacy outcomes. These were phase I or II trials with small sample sizes, limiting their power to assess relevant clinical endpoints. Although differences between the experimental and control groups did not reach statistical significance for most surrogate endpoints, there were trends suggesting potential benefits. Encouragingly, some improvements were observed in the experimental group compared to baseline, providing support for future research. However, the two most recently published studies, the DANISH^46^ and SCIENCE^44^ trials, while reaffirming safety, had disappointing results in terms of efficacy. The use of a standardized allogeneic cell product may have impacted their results.

Multiple mechanisms may underlie the potential benefits of ADSCs in ischemic heart disease. The release of paracrine factors, such as proangiogenic or antiapoptotic cytokines, may contribute to improved vascularization and reduced scar formation. Additionally, a smaller fraction of ADSCs can also differentiate into cardiomyocytes, but their relevance for regeneration of myocardial tissue has never been demonstrated.^48^

Other types of mesenchymal stem cells have been investigated in patients with ischemic heart disease. A recent phase III randomized clinical trial assessed the use of bone marrow-derived mesenchymal precursor cells in patients with advanced heart failure, predominantly of ischemic origin. Although the trial did not meet its primary and secondary endpoints, post-hoc analyses demonstrated potential benefits in certain subgroups, such as patients with elevated high-sensitivity C-reactive protein.^49^Another trial showed sustained improvements, over 12 months, in regional myocardial ischemia and coronary flow reserve associated with bone marrow cell transplantation in chronic ischemic patients.^50^ Previous trials have demonstrated the safety and potential benefits of these cell therapies.^51-53^ Umbilical cord and Wharton’s jelly-derived mesenchymal stem cells have also shown promising results in patients with ischemic heart disease.^54,55^ Although no clinical studies have directly compared different types of mesenchymal stem cells, they have exhibited similar safety profiles and benefits. Factors such as cost and the challenges associated with harvesting and expanding these cells may influence the choice of the most suitable cell type for treatment.

From the clinical standpoint, there is a great need for new therapies in patients with ischemic heart disease. Despite progress in surgical techniques and percutaneous coronary intervention technologies, there is a relevant number of patients with angina who are either suboptimal candidates for revascularization or in whom revascularization is not feasible.^22^ Perhaps the biggest demand for new therapies is for patients who develop heart failure with reduced ejection fraction after an ischemic event. They have worse prognosis when compared to those with heart failure from other etiologies and often progress to advanced disease, with refractory symptoms despite optimal therapy.^23^ In these cases, the therapeutic options are limited to left ventricular assist devices, which are expensive and not available in many countries, or heart transplant, which depends on organ availability and has multiple pre-requisites for candidate patients. This scenario is reflected by ADSC trials, which majorly included symptomatic patients with reduced ejection fraction. However, this subgroup of patients often has highly remodeled chronic myocardial disease with large fibrotic areas, and perhaps eventual improvements in vascularization or repair with ADSCs might come too late in the natural history of the disease. Possibly, the sweet spot lies closer to the acute ischemic event, where there is larger potential to reduce scar formation and prevent remodeling.

One of the main limitations in the field is the lack of standardization of cell preparations, delivery methods (e.g., intracoronary infusion versus intramyocardial injection), and efficacy outcomes (e.g., LVEF, reduction in infarction size, increased myocardial perfusion, or exercise tolerance), which poses a major challenge in assessing the safety and efficacy of cell-based therapies in clinical trials. Establishing standardized protocols for cell handling and delivery will expedite the translational process and facilitate larger clinical trials to evaluate this promising therapeutic strategy. Another limitation of this study is lack of a metanalysis or quantitative data synthesis. However, considering the great heterogeneity of the studies, we presumed that a quantitative approach to data synthesis could lead to misleading conclusions and therefore opted for a narrative synthesis.

## Conclusions

Based on small studies of patients with ischemic heart disease, ADSC injection appears to be safe and showed some preliminary beneficial effects. Further exploration is warranted to target diminishing the inflammatory and fibrotic responses, and to improve the cardiac microcirculation function in these patients. While the intervention seems feasible, safe, and promising, larger clinical trials are necessary to evaluate the efficacy of ADSCs in patients with ischemic heart disease.
